# Ferroptosis inhibition by deferiprone, attenuates myelin damage and promotes neuroprotection in demyelinated optic nerve

**DOI:** 10.1038/s41598-022-24152-2

**Published:** 2022-11-16

**Authors:** Atefeh Rayatpour, Forough Foolad, Motahareh Heibatollahi, Khosro Khajeh, Mohammad Javan

**Affiliations:** 1 Department of Physiology, Faculty of Medical Sciences, Tarbiat Modares University, Tehran, Iran; 2 Institute for Brain and Cognition, Tarbiat Modares University, Tehran, Iran; 3 Department of Pathology, School of Medicine, Shahid Beheshti University of Medical Sciences, Tehran, Iran; 4 Department of Nanobiotechnology, Faculty of Biological Sciences, Tarbiat Modares University, Tehran, Iran; 5 Department of Biochemistry, Faculty of Biological Sciences, Tarbiat Modares University, Tehran, Iran

**Keywords:** Pharmacology, Multiple sclerosis, Neurodegenerative diseases, Neurophysiology, Cell death in the nervous system, Cellular neuroscience, Gliogenesis, Myelin biology and repair

## Abstract

Multiple sclerosis (MS) is a chronic inflammatory disease, which leads to focal demyelination in the brain and spinal cord. Studies showed that iron released during the course of myelin breakdown exacerbates tissue damage, which is in agreement with the features of iron-dependent cell death, ferroptosis. Here, we aimed to investigate the possible contribution of ferroptosis in the demyelinated optic nerve, and to explore the effectiveness of ferroptosis inhibitor, deferiprone (DFP), on the extent of demyelination, inflammation and axonal damage. For this purpose, focal demyelination was induced by injection of lysolecithin (LPC), into the optic nerve of male C57BL/6J mice. Afterward, optic nerves were harvested at different time points from as early as 6 h up to 7 days post-LPC injection. Next, to evaluate the effectiveness of DFP two groups of animals received daily intraperitoneal injection of DFP for 3 or 7 continuous days. Vehicle groups received saline. Iron deposition was observed at different time points post-LPC injection from 6 h to 7 days post injection. Examining ferroptosis markers showed a significant reduction in glutathione content along with increased level of malondialdehyde and upregulated ferroptosis marker genes at early time points after injection. Besides, DFP treatment during the inflammatory phase of the model resulted in decreased microgliosis and inflammation. Reduced demyelination, microgliosis and astrogliosis was shown in mice that received DFP for 7 days. Moreover, DFP protected against axonal damage and retinal ganglion cells loss. Our results suggest the possible contribution of ferroptosis pathway in the process of demyelination. The therapeutic strategies targeting iron deposition, e.g. DFP treatment might thus represent a promising therapeutic target for patients with MS.

## Introduction

Multiple sclerosis (MS) is a chronic inflammatory disease of the central nervous system (CNS), which leads to focal lesions in the white and gray matters of the brain and spinal cord, characterized by primary demyelination with a variable extent of axonal loss^1^. There is currently no effective cure for MS, and our understanding of the pathological events that contribute to MS is limited.

Oxidative stress, mitochondrial dysfunction, disruption of energy homeostasis and dysregulation of iron metabolism are important factors involved in plaque formation and neurodegeneration in white and gray matter lesions^2,3^. In healthy brain, iron is mainly stored in oligodendrocytes and myelin sheets, which acts as a cofactor for enzymes involved in myelin maintenance and synthetize^4–6^. Upon demyelination, iron released from dying oligodendrocytes, generates reactive oxygen species (ROS)^3^. Studies have shown that oxidative damage and lipid peroxidation in acute demyelination are correlated with the amount of iron released during the course of myelin breakdown^2,7^. Liberated iron is then taken up by activated microglia and macrophages, leading to dystrophy of these cells and therefore releasing iron into the extracellular space, which initiates a secondary wave of oxidative stress^3,7^. Furthermore, iron overload in microglia and macrophages promotes an M1 phenotype, which amplifies the inflammatory response^8^. All of this suggests the detrimental role of iron in the pathogenesis of MS.

A recently introduced type of iron-dependent oxidative cell death called ferroptosis^9^, shares several features with the pathological events of the MS. First, iron deposition that triggers ferroptosis^9^, is reported to be present in the rims and center of active demyelinating lesions in patients postmortem tissues^3^. Second, abnormal mitochondrial morphology and decreased expression of ferroptosis regulator, gluthation peroxide 4 (GPX4), which is known as the only enzyme capable of reducing lipid peroxides, are reported in experimental autoimmune encephalomyelitis (EAE) and MS lesions^10^. Third, lipid peroxidation generated from iron-catalyzed oxidation of poly-unsaturated fatty acids, is one of the important features of MS lesions and ferropotosis as well. Given that ferroptosis features including iron deposition, lipid peroxidation, mitochondrial morphological changes and dysfunction, are all present in demyelinating lesions^10,11^, we hypothesized that ferroptosis pathway may play a crucial role in demyelinating lesions.

Deferiprone (DFP), a potent iron chelator, is approved by Food and Drug Administration (FDA) and used for the treatment of diseases with iron overload^12^. DFP treatment in the active phase of the EAE model dramatically reduced clinical symptoms and inhibited T lymphocyte infiltration and proliferation^13^. Another study also showed that dextrase 1 gene deletion (an important gene involved in iron export) combined with DFP treatment maintained rat vision and prevented retinal ganglion cells (RGCs) loss and axonal degradation in the EAE optic neuritis model^14^. While the neuroprotective effect of DFP has been well studied, its underlying mechanisms remain elusive. Deferiprone also has protective effects against neuronal death induced by hydrogen peroxide, amyloid beta, 1-methyl-4-phenylpyridinium (MPP^+^) and ferric nitrilotriacetate (FeNTA), in vitro^15^. In addition, low-dose of this iron chelator, had protective effects in the Parkinson's animal model^16^. In the animal model of Alzheimer's disease, treatment with DFP reduced memory impairment and prevented iron deposition and amyloid beta accumulation^17^. In another study, deferiprone was shown to reduce mitochondrial iron in the Huntington's disease model and subsequently reduced lipid peroxidation and resulted in improved functional recovery^18^. Additionally, the protective effects of DFP has been well studied in different models of retinal degeneration^19–23^.

Although iron deposition has been reported to be involved in the pathogenesis of several neurodegenerative diseases, the contribution of ferroptosis in demyelination condition as well as the effect of DFP on iron deposition and the extent of myelin damage has not yet been clarified. Therefore, in the present study we set out to confirm the possible contribution of ferroptosis in LPC induced demyelination by investigating alterations of ferroptosis related markers. Furthermore, we showed that removing excess iron via DFP could preserve myelin.

## Results

### Iron deposition was observed in optic nerve following LPC injection

**Figure 1:**
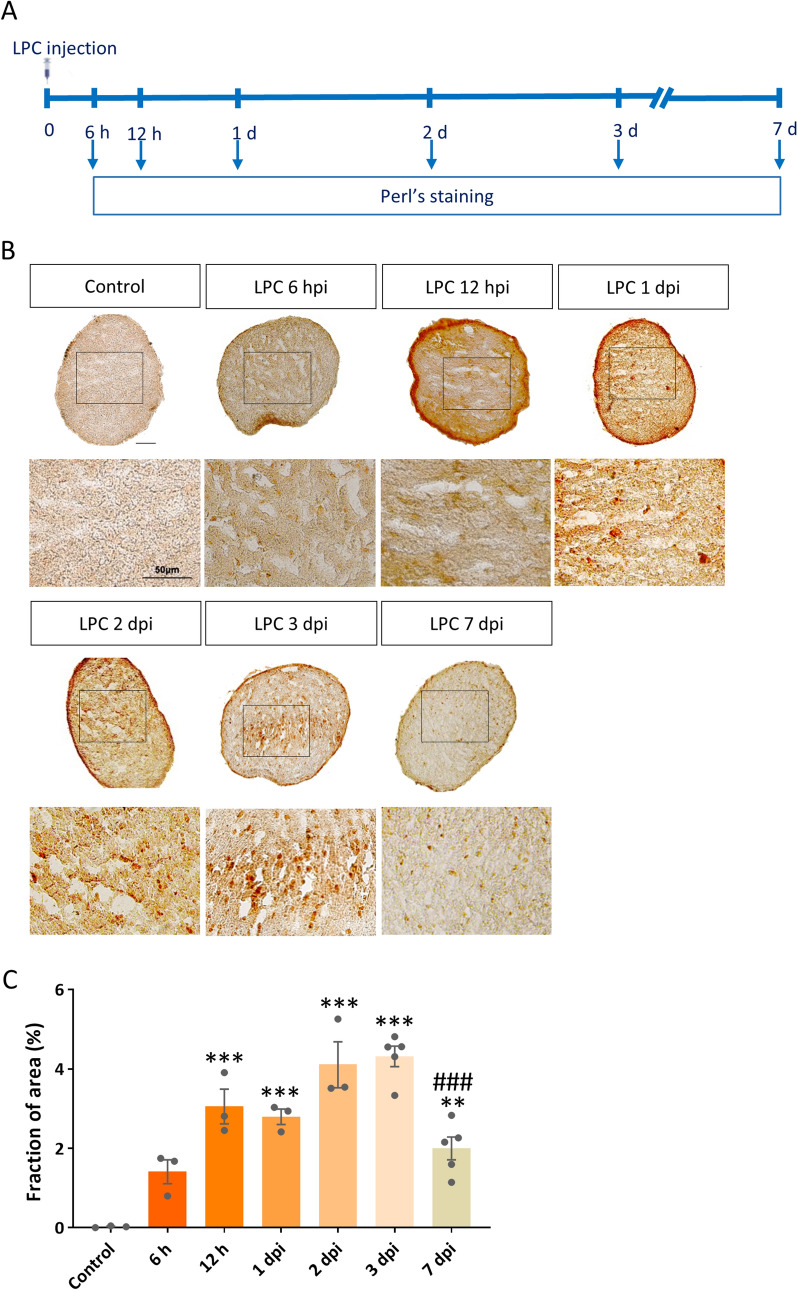
Iron accumulation in optic nerve following LPC injection. (**A**) Schematic illustration shows the time line of LPC injection and Perl’s staining in the experimental setting used. (**B**) DAB-enhanced Perl’s staining shows iron deposition in optic nerve sections at different time points starting at 6 h after LPC injection. (**C**) Quantification of iron positive fraction of area. Data represent mean ± SEM, one-way ANOVA with Tukey's multiple comparison post-test, n = 3–5 mice per group. **p < 0.01, ***p < 0.001 versus control, ^###^*p* < 0.001 versus 3 dpi. Scale bar = 50 µm. hpi: hour post-injection.

Since iron deposition is the key factor that triggers ferroptosis^9^, we first evaluated whether iron overload and other ferroptosis markers are present in the LPC model of MS. To explore this question in more details, we injected LPC into the optic nerve of mice and assessed the time course of local iron deposition from as early as 6 h up to 7 days post injection (dpi) (Fig. 1A). Seven days post LPC was reported to be time point of max demyelination occurrence^24^. The fraction of iron‐positive area was increased 6 h after LPC injection and continued to increase over the time and reached the maximum level at 2 and 3 dpi (*p* < 0.001 for 12 h and day 1–3, *p* < 0.01 day 7, vs. control), then followed by a significant reduction at 7 dpi (*p* < 0.001 vs. 3 dpi, Fig. 1B,C). Due to the key role of iron overload in ROS production and subsequent lipid peroxidation, we next assessed the ferroptosis pathway at different time points following LPC injection.

### Lipid peroxidation and alteration of ferroptosis‐related genes were detected in the injured optic nerve

**Figure 2:**
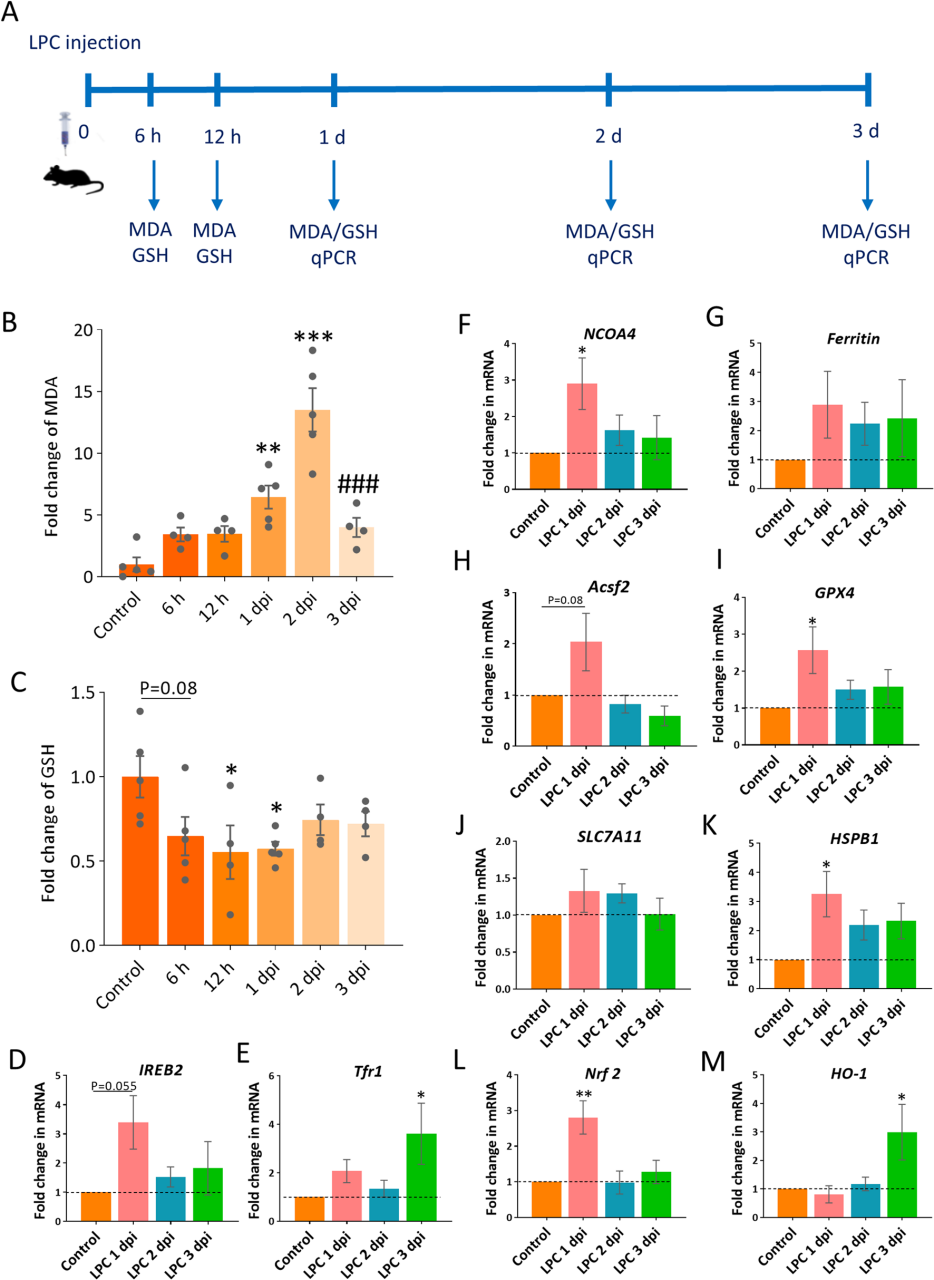
Evaluation of ferroptosis markers following LPC injection. (**A**) Schematic illustration shows the time line of LPC injection and tissue sampling for biochemical and molecular assessments. (**B**) Spectrophotometric detection of malondialdehyde (MDA) and (**C**) glutathione (GSH) at different time points after LPC injection. Data represent mean ± SEM, one-way ANOVA with Tukey's multiple comparison test, n = 4–5 mice per group. *p < 0.05, **p < 0.01, ***p < 0.001 versus control, ^###^*p* < 0.001 versus 2 dpi. (**D**–**M**) Ferroptosis related genes expression at different time points following LPC injection. One-way ANOVA with Dunnett's multiple comparisons test, Data represent mean ± SEM, n = 4–5 mice per group (3 animals pooled for each sample; total 12–15 animals used for each time point), *p < 0.05, **p < 0.01 versus control.

The iron accumulation may lead to iron catalyzed Fenton reaction, which generates free radicals, thus further oxidizing lipids^25^. Malondialdehyde (MDA) is an end product of polyunsaturated fatty acids peroxidation^26^. High level of free radicals, therefore, leads to MDA overproduction, which is commonly known as an essential hallmark of ferroptosis. To explore whether iron deposition following LPC injection may lead to lipid peroxidation, we assessed the MDA concentration at 6 and 12 h, 1, 2 and 3 days after LPC injection (Fig. 2A). Our results showed that MDA was elevated at 6 and 12 h after LPC injection, 3.42- and 3.45-fold change, respectively. This increase remained elevated at 6.45 fold at 1 dpi (*p* < 0.01), followed by a greater increase at 2 dpi (13.5 fold, *p* < 0.001). Afterward, MDA concentration decreased significantly at 3 dpi (*p* < 0.001 vs. 2 dpi, Fig. 2B).

Another key feature of ferroptosis is insufficient level of glutathione that scavenge lipid radicals^27^. We therefore, assessed GSH concentration to evaluate the antioxidant capacity of the cells at different time points post LPC injection. At 6 h after LPC injection, there appeared to be a small reduction in GSH level although it was not statistically significant (*p* = 0.08). However, compared to Control, the GSH concentration was significantly reduced at 12 h and 1 dpi (*p* < 0.05) and increased slightly at 2 and 3 dpi (Fig. 2C). This result was consistent with the increased in MDA concentration, since GPX4 activity and GSH directly remove lipid reactive oxygen species (lipid ROS) and protect cells against ferroptosis^27^.

We also assessed the mRNA levels of the ferroptosis‐related genes. Our results showed that, mRNA expression of several markers of ferroptosis, including Iron response element B2 (*IREB2*), Nuclear receptor coactivator 4 (*ncoa4*), transferrin receptor 1 (*tfr1*), acyl-CoA synthetase family member 2 (*Acsf2*) and heme oxygenase-1 *(HO-1)* are increased at early time points after LPC injection (Fig. 2D–M). *IREB2*, the main transcription factor of iron metabolism, showed upregulation at 1 dpi (*p* = 0.055, Fig. 2D). However, the expression of its downstream target, *tfr1*, which is recently introduced as a specific marker for ferroptosis^28^, reached the peak level at 3 dpi (*p* < 0.05, Fig. 2E). *ncoa4*, which is a cargo receptor that mediates ferritin degradation (ferritinophagy), appeared to be increased at 1 dpi (*p* < 0.05, Fig. 2F). The expression of ferritin showed non-significant upregulation at all time-points (Fig. 2G). Furthermore, the expression of *Acsf2*, another hallmark of ferroptosis, that have role in lipid metabolism was increased (ANOVA, F (3, 15) = 3.973, *p* < 0.05, post-hoc: *p* = 0.08 for 1 dpi vs. control, Fig. 2H).

Upon demyelination, the *GPX4* expression significantly increased at day 1 after induction of model (*p* < 0.05); however, this elevation did not continue in the following next days (Fig. 2I). The expression of *SLC7A11*, Cystine/glutamate antiporter xCT, that plays an important role in the transport of essential amino acids for glutathione synthesis, did not change in measured time points (Fig. 2J). We also checked the level of ferroptosis inhibitor, *HSPB1*, which showed an upregulation at 1 dpi (*p* < 0.05), however, this elevation did not continue in the following next days (Fig. 2K). We also checked the level of *(Nrf2)*, a negative ferroptosis regulator, which showed a significant increase at 1 dpi (*p* < 0.01), while did not continue in the next following days (Fig. 2L). One important downstream molecule in Nrf2-related pathway, heme oxygenase 1 *(HO-1)*, showed a significant elevation at 3 dpi (*p* < 0.05, Fig. 2M). Even though, Nrf2 is an important molecule in cellular defense pathway against oxidative stress, expression of HO-1 mediates degradation of heme to Fe^2+^, carbon monoxide and biliverdin, which increases the level of labile iron pool and likely enhances the ferroptosis^29^. Collectively, key factors in ferroptosis pathway, including the GSH, MDA, IREB2, Tfr1, NCOA4 and HO-1 were detected in LPC induced demyelination. Thus, these data suggests that iron released following LPC injection as well as the insufficient antioxidant activity might induce lipid peroxidation.

### DFP decreased iron deposition in demyelinated optic nerve

**Figure 3:**
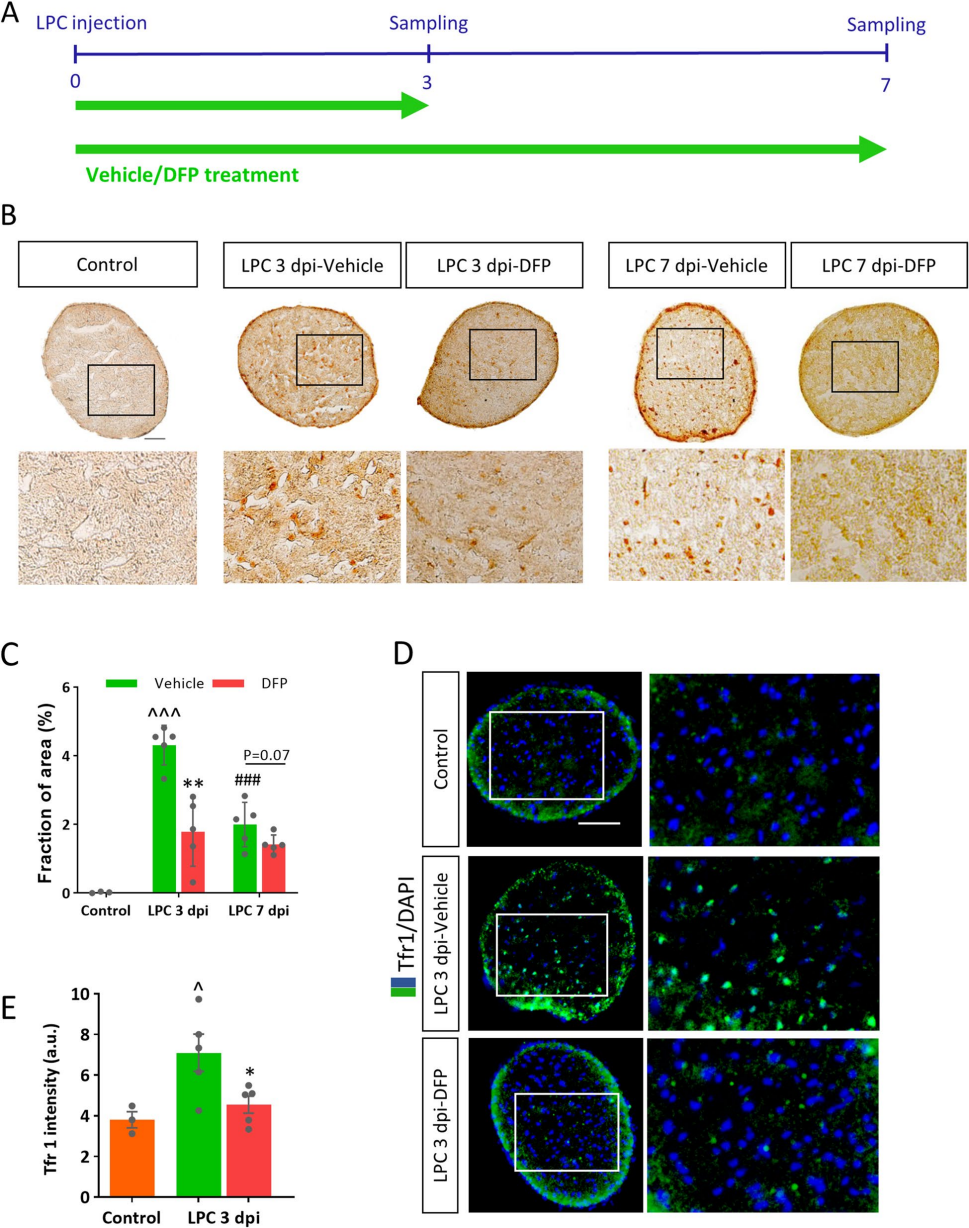
Deferiprone (DFP) decreased iron deposition in demyelinated optic nerve. (**A**) Experimental timeline of LPC injection and DFP treatment. DFP (10 mg/kg twice per day) or vehicle were injected intraperitoneally for 3 continues days. (**B**) Increased iron deposition was observed on 3 and 7 dpi as revealed by DAB enhanced Perl’s staining, while continuous DFP administration reduced iron deposition. (**C**) Quantification of iron positive fraction of area (**D**) Immunostaining against Tfr1, counterstained with DAPI in vehicle and DFP treated mice 3 days post LPC injection. (**E**) Quantification of Tfr1 immunoreactivity showed significant reduction in Tfr1 intensity in the optic nerve of DFP treated mice at 3 dpi. Data represent mean ± SEM, Student's t-test, n = 3–5 mice per group. *p < 0.05, **p < 0.01 versus vehicle at the same group, ^##^*p* < 0.01 versus 3dpi-vehicle, ^*p* < 0.05, ^^^*p* < 0.001 vs control. Scale bar = 50 µm.

Compelling evidence revealed that iron accumulation potentiates inflammatory response via activating innate immune system^30^. Given that lipid peroxidation and peak of iron deposition were detected within the early stage, which corresponds to the inflammatory phase of LPC model, we asked whether deferiprone, an iron chelator, could prevent inflammation via removing excess-deposited iron. Furthermore, in LPC model the myelin damage continues for several days after the initial insult, reach to maximum level on day 7 and leads to gliosis. We thus hypothesize that deferiprone may also reduce the severity of damages by reducing inflammation and gliosis. To address these questions in more details, we verified the impact of DFP on the iron deposition. The iron content was determined by DAB enhanced Perl’s staining after 3 or 7 days of DFP treatment (Fig. 3A). DAB enhanced Perl’s staining confirmed that the iron deposition was reduced significantly by DFP treatment (*p* < 0.01, *p* = 0.07, respectively). These all suggested that DFP was able to efficiently remove the excess iron in optic nerve following LPC-induced demyelination (Fig. 3B,C). In order to introduce specific marker to selectively detect ferroptotic cells in tissues, Feng et al. found the transferrin receptor as a specific marker following a high quantity screening^28^. Therefore, we checked the level of Tfr1 expression in optic nerve following LPC injection and asked whether DFP treatment might decrease Tfr1 (Fig. 3D,E). Immunostaining against Tfr1 was carried out at 3 dpi. As representative images showed, the intensity of Tfr1 immunoreactivity was reduced by DFP (Fig. 3D). The quantitative data proved that DFP ameliorated the Tfr1 immunoreactivity (*p* < 0.05, Fig. 3E).

### DFP attenuated inflammation, microglial activation and astrogliosis

**Figure 4:**
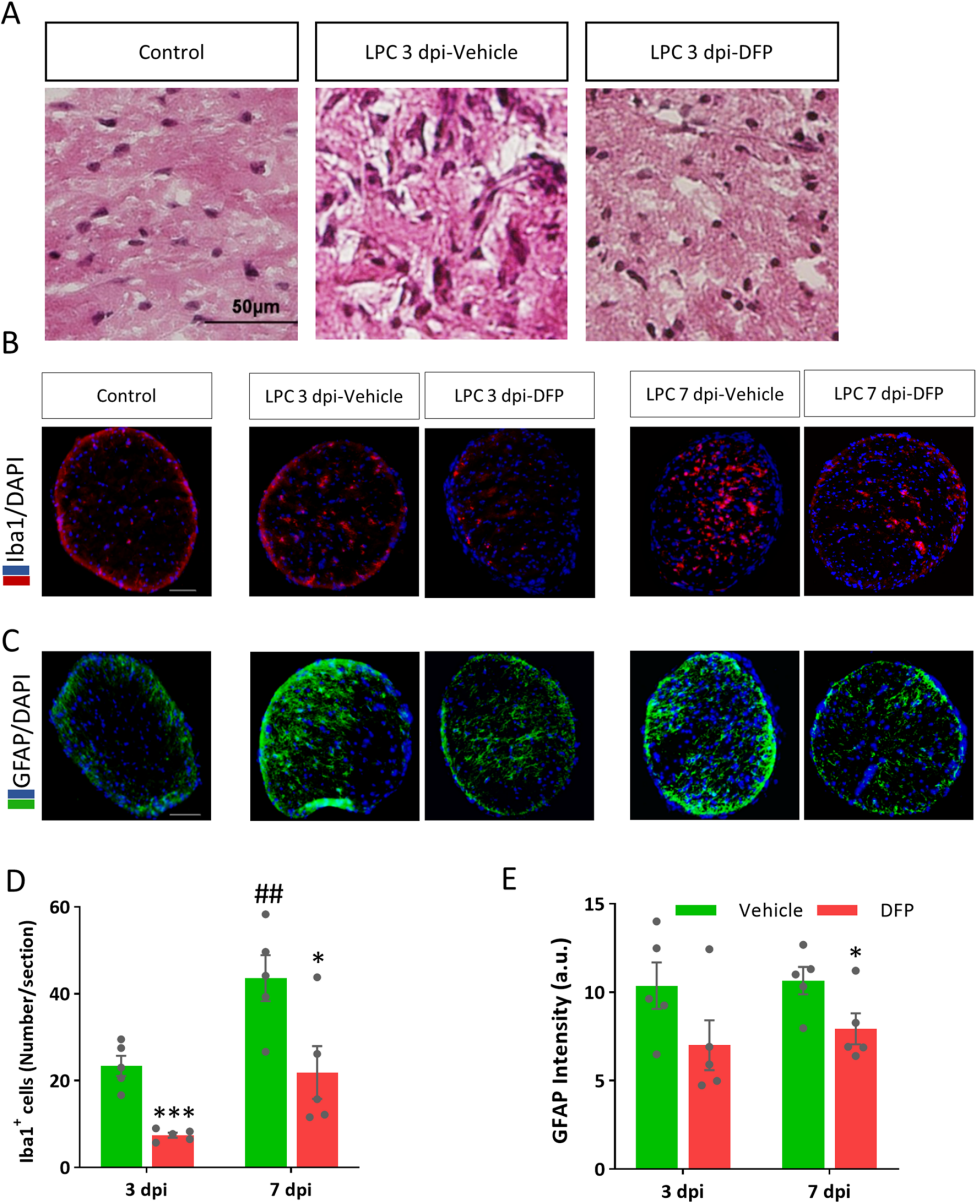
Deferiprone (DFP) attenuated inflammation and gliosis in demyelinated optic nerve. (**A**) Representative images of transverse sections from the optic nerve at 3 dpi that stained with hematoxylin and eosin. (**B**) Immunostaining against Iba1, the microglia marker, counterstained with DAPI in vehicle and DFP treated mice on 3 and 7 dpi. (**C**) Immunostaining against GFAP, as the astrocyte marker, counterstained with DAPI in vehicle and DFP treated mice 3 or 7 days post LPC injection. (**D**) Quantification of the number of Iba1^+^ cells showed that DFP ameliorated microglia activation in the optic nerve following LPC insult at 3 or 7 dpi. (**E**) Quantification of GFAP immunoreactivity showed non-significant reduction in astrogliosis in the optic nerve following LPC insult at 3 dpi along with significant decrease at 7 dpi. Data represent mean ± SEM. Student's t-test. *p < 0.05, ***p < 0.001 versus vehicle at the same group, ^##^*p* < 0.01 versus LPC 3dpi-vehicle, n = 5 mice per group, Scale bar = 50 µm.

We assessed the effect of DFP on the infiltration of peripheral immune cells such as neutrophil and lymphocytes using H&E staining of sections obtained from animals scarified at 3 dpi. H&E staining demonstrated no differences among groups regarding the infiltration of peripheral immune cells; however, the total inflammation score, determined by the pathologist, was reduced in DFP treated animals (Fig. 4A).

To evaluate the inhibitory effect of DFP on microgliosis, immunostaining against Iba1, as a microglia marker, was carried out on 3 and 7 dpi. As representative micrographs show, the number of activated microglia was increased on 3 and 7 dpi while it was reduced by DFP (Fig. 4B). Furthermore, data quantification showed that DFP treatment significantly ameliorated the number of Iba1 positive cells on both 3 (*p* < 0.001) and 7 dpi (*p* < 0.05, Fig. 4D).

To explore the effect of DFP on astrogliosis, immunofluorescence staining was used to show the extent of GFAP reactivity. Gliosis was obvious surrounding the damaged area on 3 and 7 dpi. As data quantification showed, 3 days of DFP treatment led to a non-significant reduction in astrogliosis; however, it significantly reduced the GFAP immunoreactivity following 7 days (*p* < 0.05, Fig. 4C,E).

### Treatment with DFP decreased the myelin loss

**Figure 5:**
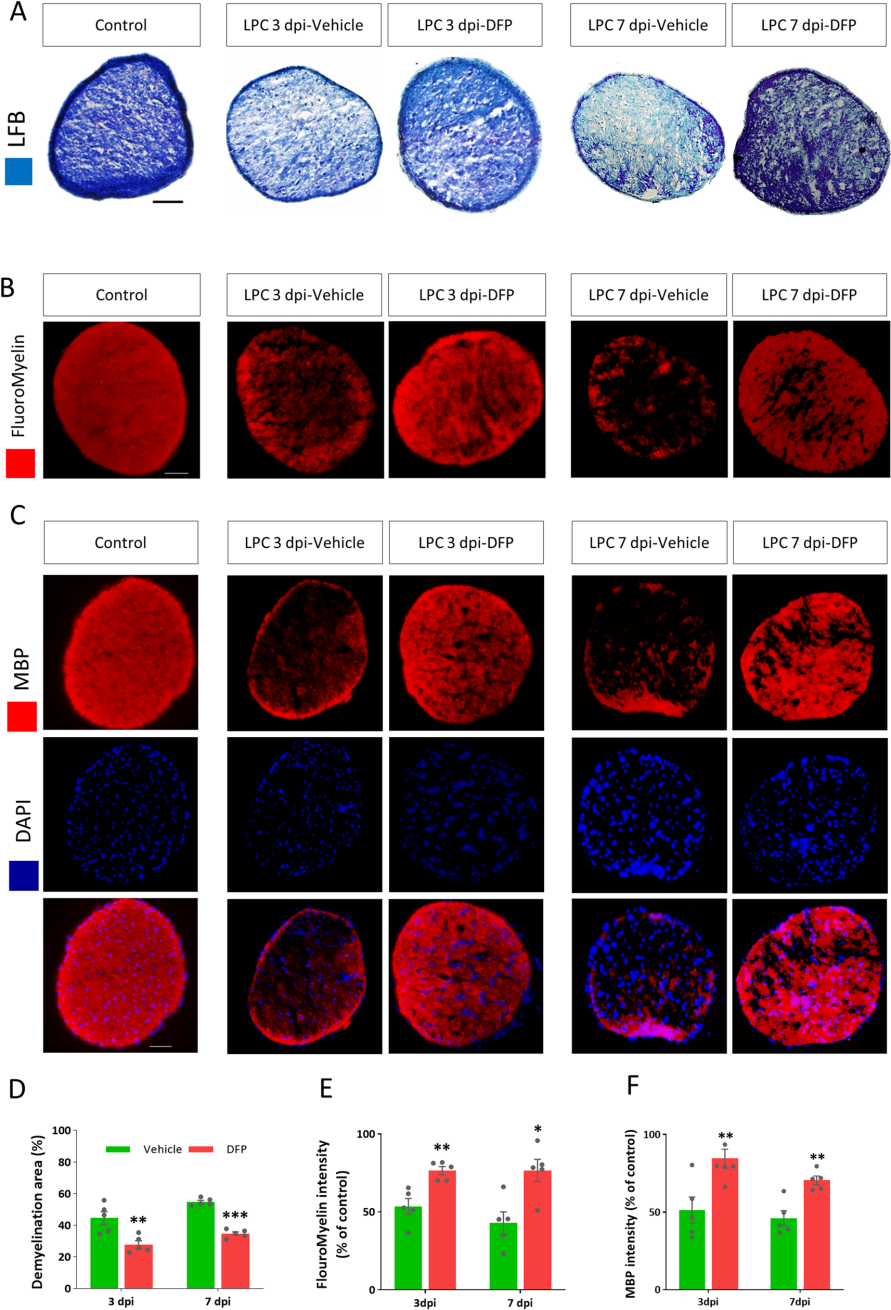
Treatment with Deferiprone (DFP) decreased an extensive loss of myelin in the optic nerve. (**A**) Representative micrographs of optic nerve prepared from intact and LPC-injected mice treated with DFP or vehicle for 3 or 7 days, stained with LFB and crysel violet. (**B**) Representative images of FluoroMyelin stained sections of intact and LPC-injected mice treated with DFP or vehicle for 3 or 7 continues days. (**C**) Immunostaining against MBP counterstained with DAPI in intact and LPC-injected mice treated with DFP or vehicle 3 or 7 days. (**D**) Quantitative analysis of the LFB-stained sections indicated reduced demyelination in mice treated with DFP. The bar graph shows the percentage of demyelination area versus the total area of the transverse section of optic nerve. (**E**) Quantitative analysis of FluoroMyelin intensity, calculated as the percent of the FM reactivity in intact group. (**F**) Quantitative analysis of MBP intensity showed significant differences between vehicle and DFP treated groups on 3 and 7 dpi. Data represent mean ± SEM. Student's t-test. *p < 0.05, **p < 0.01, ***p < 0.001 versus vehicle at the same group, n = 5 mice per group, Scale bar = 50 µm.

To address whether treatment with the ferroptosis inhibitor, DFP, decreases an extensive myelin loss caused by LPC injection, LFB staining was used to measure the extent of demyelination on 3 and 7 dpi (Fig. 5A). In DFP-treated groups, demyelinated area was significantly reduced compared to vehicle groups at 3 (*p* < 0.01) and 7 dpi (*p* < 0.001, Fig. 5D).

We also checked the severity of demyelination via FluoroMyelin (FM) staining (Fig. 5B). Quantitative data showed that FluoroMyelin intensity in DFP groups was significantly higher than vehicle-treated animals on both 3 (*p* < 0.01) and 7 (*p* < 0.05) dpi (Fig. 5E).

As a more specific staining method, immunostaining against myelin antigen (MBP) confirmed the results of LFB and FM staining (Fig. 5C). The quantification of data indicated that immunoreactivity against MBP was significantly increased in DFP-treated mice on 3 and 7 dpi (both *p* < 0.01, Fig. 5F).

### DFP decreased axonal damage and retinal ganglion cell loss

**Figure 6:**
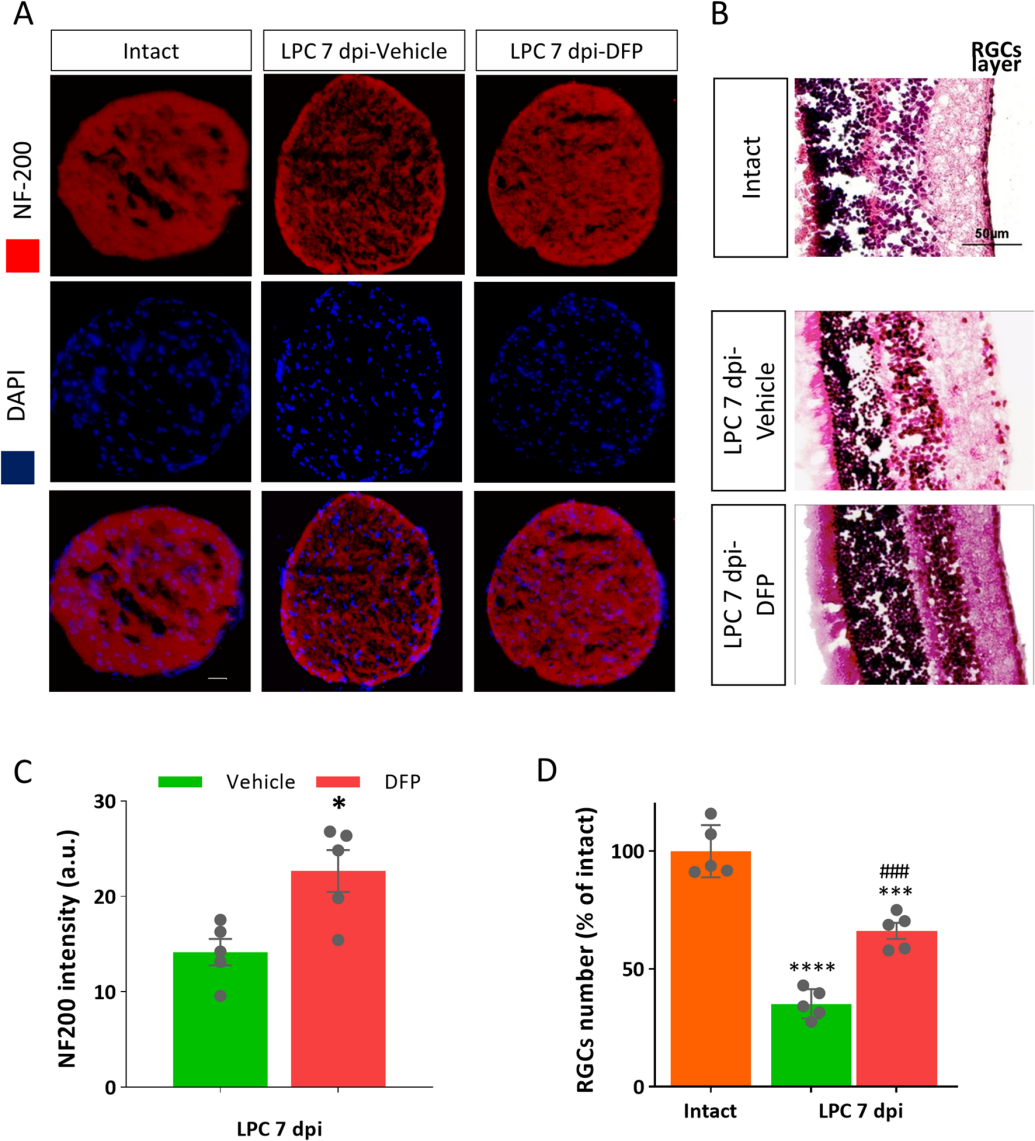
Deferiprone decreased retinal ganglion cell (RGC) loss in demyelinated optic nerve. (**A**) Immunostaining against NF-200, as the axonal marker, counterstained with DAPI in vehicle and deferiprone (DFP) treated mice on 7 dpi. (**B**) H&E staining demonstrated that DFP protects against the loss of RGCs. (**C**) Quantification of the NF-200 immunoreactivity showed that DFP protected against axonal damage at 7dpi. *p<0.05 versus vehicle group, Student's t-test. (**D**) Quantification of the number of RGCs. Data represent mean ± SEM. One-way ANOVA with Tukey's multiple comparisions test, ***p< 0.001 versus Intact group, ###*p*<0.001 versus LPC 7dpi-vehicle, n = 5 mice per group, Scale bar = 50 µm.

Since the LPC injection in optic nerve is associated with some degree of axonal damage^31^, we examined the effect of DFP on axonal damage using immunostaining against NF-200. We also asked whether this axonal damage leads to retinal ganglion cells (RGCs) loss and, if so, whether DFP could preserve them. Our results showed significant increase on NF-200 immunoreactivity in DFP-treated mice compared to vehicle at 7 dpi (*p* < 0.05, Fig. 6A,C), suggesting the neuroprotective effect of DFP in this experimental setting. We then checked the number of RGCs using H&E staining of the eye globe. Our results indicated that DFP significantly preserved RGC loss on 7 dpi (*p* < 0.001, Fig. 6B,D).

## Discussion

Ferroptosis is a newly discovered iron dependent regulated cell death that has received much attention in recent years. Increasing evidence have demonstrated the role of ferroptosis in the pathogenesis of neurodegenerative disease^32^. A recent study has shown that the expression of ferroptosis inhibitor, GPX4, was reduced in MS and EAE lesions^10^. Furthermore, a very recent study has shown that using ferroptosis inhibitor, Liproxstatin-1, or reducing ACSL4 expression reduced neuroinflammation and neural death in EAE model^33^. However, its role in demyelinating conditions has not been reported. In the present study, we found that upon demyelination induction with LPC in mouse optic nerve, iron accumulated at early time points after the injury. Furthermore, the level of MDA demonstrating lipid peroxidation, was increased. Additionally, GSH level was diminished along with upregulation of ferroptosis related genes at 1–3 days after induction of demyelination. This time point covers the beginning of demyelination processes and is correspond to the severe inflammatory phase following LPC^24^.

Iron, the key factor that triggers ferroptosis^9^, is accumulated in several neurodegenerative diseases such as Alzheimer’s disease, Parkinson’s disease, Amyotrophic lateral sclerosis (ALS) and MS^32^. In the context of MS; however, one important source of iron released in the extracellular space is dying oligodendrocytes and myelin debris^3^. Iron is a required co-factor for a variety of enzymes involved in the maintenance and production of myelin^5^. In liberated form; however, excessive free iron (Fe^2+^) may produce hydroxyl radicals (.OH) via Fenton chemistry^25^. This process initiates lipid peroxidation, which is an essential hallmark of ferroptosis. Iron deposition might also promote oxidative stress, inflammation and glutamate toxicity, which exacerbates cytotoxicity and neurodegeneration^7^. Moreover, several studies reported that the level of iron deposition positively correlates with the disease progression in patients with MS^34–37^.

Our results showed a temporal profile of iron accumulation changes. Iron deposition was observed from 6 h post-LPC and continued to elevate over time within 3 days after LPC injection. Then, there was a significant reduction in iron deposition at 7dpi, which is considered as the peak of demyelination. In LPC model, the measured extent of demyelination is the cumulative result of all pathological events including inflammation, iron accumulation and subsequent oxidative stress happened in previous days. This may explain why the extent of demyelination reached the maximum amount at day 7, while iron accumulation and inflammation were at the maximum level on previous days.

In this study, in addition to the iron deposition during the early period of LPC demyelination, alterations were also seen in the expression of markers of ferroptosis including increased *IREB2*, *TfR1*, *Acsf2*, *NCOA4* and *HO-1*. Iron homeostasis is regulated by the balance between iron uptakes, storage, utilization and efflux. We observed upregulation of *IREB2* and its downstream target, *Tfr1*, at 1 and 3 dpi, respectively. *IREB2* is reported to be tightly associated with the iron regulation in CNS^38^. Moreover, increased expression of Tfr1 was also detected by immunostaining at 3 dpi. Increasing evidences have reported that transferrin import via transferrin receptor is essential for the occurrence of ferroptosis^28,39^. Recently, in order to find a reliable approach to selectively detect ferroptotic cells in tissues, Feng et al. screened 4750 monoclonal antibodies against the cells induced to undergo ferroptosis via erastin. Additional screening revealed several candidates including Tfr1 and MDA. They have validated these antibodies to detect ferroptotic cells in xenograft cancer models^28^. Furthermore, Gao et al. showed that both transferrin import and glutaminolysis are required for ferroptosis. Thus, it is suggested that Tfr1 and glutamine metabolic pathway play crucial role during ferroptotic cell death^39^. Here, the increased expression of Tfr1 at 3 dpi, as revealed by immunostaining suggests the possible contribution of ferroptosis during LPC-demyelination.

Ferritin stores excess iron, by holding up to 4500 atoms of iron, and prevents iron to be involved in chemical reactions^40^. Normally, if iron is needed, ferritin is degraded, which is termed ferritinophagy and the iron is released^41^. A recent study has shown that autophagy is associated with ferroptosis via degradation of ferritin in autophagosomes^42^. The expression of molecules involved in ferritinophagy is therefore, another characteristic of ferroptosis. In our study, an increase in NCOA4, a cargo receptor that binds to ferritin and shuttles it into autophagosomes^41^, was seen at the first day after induction of demyelination, suggesting the loss of ferritin and releasing further iron. In addition, our results showed a non-significant trend to increase ferritin at 1, 2 and 3 dpi, which may be considered as a compensatory response to iron overload and ferritinophagy. Taken together, iron deposition revealed by Perl’s staining as well as dysregulation of iron homeostasis at early time points after LPC injection suggests their possible role in inflammation and demyelination phenomenon. Consistence with our results, a recent study confirmed ferroptosis contribution to the loss of oligodendrocytes in the cuprizone model^11^.

We next observed elevated MDA concentration after LPC injection, which reached the maximum level at 2 dpi, which was coincided with the timing of iron deposition. This high MDA level was followed by a rapid restoration at 3 dpi, suggesting rapid oxidation of this highly toxic molecule to final products of H2O2 and CO2^26,43^. The extent of lipid peroxidation and MDA accumulation is reported to be correlated with inflammation, demyelination and neurodegeneration in patients with MS^44^. In agreement with our results, a recent study reported elevation of lipid peroxidation markers including MDA and 4-HNE during the course of cuprizone induced demyelination^11^. Furthermore, the expression of ACSF2 that has role in lipid metabolism was increased. ACSF2 mediates synthesis of specific lipid precursors required for ferroptosis^9^.

Another key regulator of ferroptosis are related to molecules involved in the anti-oxidant capacity of the cells. The key molecules involved in this pathway are GPX4, the only enzyme that diminishes lipid peroxides to the alcohol via reducing GSH and SLC7A11 (xCT), an antiporter that exchanges glutamate and cystine to produce GSH^27^. We observed a mild reduction in GSH 6 h after LPC injection. However, the GSH concentration was significantly reduced at 12 h and day 1. This result is consistent with the increase in MDA concentration, since GPX4 activity via GSH directly removes lipid reactive oxygen species (lipid ROS). Indeed, GPX4 catalyzes the reaction between GSH and lipid ROS, therefore protecting cells against ferroptosis^27^. Depletion of GSH observed at early time points after LPC injection could be due to the direct effect of iron^45,46^, since SLC7A11 expression did not change at any time points. Furthermore, reducing GSH concentration may cause GPX4 inactivation, which results in increased lipid peroxidation and ferroptosis^27^. Although our results showed an increase in GPX4 expression on day 1, this elevation did not continue in the following days. The increase in GPX4 expression following the induction of demyelination in our study is in contrast to the findings of Hu et al. in the EAE model^10^ and Jhelum et al. in the cuprizone model^11^, which reflects that pathological processes involved in different models, may lead to different expression profiles. However, this increase in GPX4 expression could be the initial compensatory response to injury, since GPX4 is the main key endogenous enzyme that inhibits lipid peroxidation.

Another ferroptosis regulator was Nrf2, which showed an increase at 1 dpi. Nrf2 is a molecule in cellular defensive system, which increase the resistance of the cells to ferroptosis. Its increase might reflect a compensatory response to oxidative stress. Moreover, the expression of Nrf2 could elevate the expression of HO-1 at 3 dpi. It is reported that expression of HO-1 mediates degradation of heme to Fe^2+^, carbon monoxide and biliverdin, which increase the level of free iron and might enhance ferroptosis^29^. In agreement with our results, a recent study reported the elevation of *HO-1* mRNA expression during the course of cuprizone induced demyelination^11^.

HSPB1 is another regulator of ferroptosis. In the phosphorylated form that is mediated by protein kinase C, HSPB1 stabilizes actin of the cytoskeleton resulting in reduced iron uptake and subsequent lipid peroxidation and ferroptosis^47^. Our results showed a significant increase in HSPB1 expression within 1 dpi, which is probably an initial response to LPC insult; however, this elevation was not continued in the following days.

Collectively, our data suggest that iron deposited during the demyelination period as well as deficient GSH may promote lipid peroxidation during the course of myelin breakdown. Consistent with our results, dysregulation of iron metabolism, MDA accumulation and limited ability to repair lipid damages due to GSH deficiency are previously reported in the EAE model and cuprizone induced demyelination as well as human MS lesions^10,11^. Furthermore, in the cuprizone model, loss of oligodendrocytes was reported to coincide with the timing of several ferroptosis markers expression^11^.

Since the capacity of adult CNS for repair is limited, developing new approaches that prevent demyelination or preserve myelin are highly necessary. Here, we used an animal model of demyelination induced by LPC, which leads to sequential events and the demyelination process could be studied, separately. To obtain a direct evidence that iron deposition plays role in the extension of demyelination in LPC model, we treated mice with DFP, an iron chelator, to remove the excess iron. Our results showed a significant decrease in iron deposition and demyelination in DFP treated mice at 3 and 7 dpi. However, it remains to be elucidated whether reduced iron deposition represents the only mechanism of action of DFP in this experimental setting. DFP is known as a cell-permeable iron chelator, with the efficacy to reduce the iron accumulation in the dentate nuclei of patients with Friedreich ataxia as well as dentate and caudate nuclei of patients with Parkinson’s disease^48,49^. Additionally, DFP was reported to reduce iron overload in the retina of Ceruloplasmin/Hephaestin double-knockout mice^23^. Moreover, to induce iron deficiency in substantia nigra (SN) and explore its relationship with locomotion activity, DFP was directly injected to SN, and confirmed the iron chelating efficacy of DFP within brain^50^. At the mechanistic level, DFP is reported to relocate cellular accumulated iron within cell compartments or across the cell membrane. It also carried excess iron to the extracellular transferrin for redistribution^51^, preventing its participation in oxidative stress.

Since our results showed an increase in the Tfr1 expression by LPC, we asked whether DFP could decrease Tfr1 expression. Our results showed significant decrease in Tfr1 expression in DFP treated group, that suggests the possible contribution of ferroptosis in demyelination context.

Additionally, iron accumulation and ferroptosis were reported to promote inflammatory responses via activating innate immune system^30^. Inflammation is a key player in MS and demyelination pathogenesis that peaks at days 2–3 dpi, characterized by initial edema and glial scar formation^52^, the time period that showed the increased ferroptosis markers in our study. Our results showed no differences regarding infiltration of peripheral immune cells on 3 dpi. This result is in agreement with the previous report that showed the transient response of neutrophil and lymphocytes at 6 to 12 h after LPC injection into the spinal cord^53^. At later time points; however, the microglia, macrophages and astrocytes shape the immune response^52,53^. Our results showed that DFP reduced the severity of inflammation, microgliosis and astrogliosis. Microgliosis was reduced at 3 and 7dpi, suggesting that DFP affects the inflammation at the initial steps of demyelination induced by the LPC. We also found that DFP treatment reduced the severity of microgliosis and astrogliosis at 7 dpi, suggesting myelin protection. However, it remains to be clarified whether the excess iron removal and ferroptosis inhibition represent the mechanism of the anti-inflammatory effects of DFP in the demyelinated optic nerve.

Due to the potent anti-inflammatory effects of DFP in this experimental setting, we also evaluated the extent of demyelination. Our results revealed that DFP reduced the extent of demyelination as it was evaluated by LFB and FluoroMyelin staining as well as MBP immunostaining. Reduced demyelination in DFP-treated mice at 3 and 7 dpi might be the result of myelin protection or enhanced myelin repair. Our findings were in agreement with a recent study, which showed that iron chelation with deferoxamine, another iron chelator, protects oligodendrocytes in the spinal cord injury model^54^.

Both inflammation and continues demyelination may lead to axonal damage and subsequent neuronal loss^55^. We also found that DFP had neuroprotective effect and prevent axonal damage and RGCs loss. Our results are consistence with a very recent report, which showed that DFP preserves RGCs number and protects against axonal damages in the optic nerve of EAE mice^14^. Several studies have shown the neuroprotective effects of DFP on different retinal degeneration models induced by sodium iodate, tunicamycin and light as well as Ceruloplasmin/Hephaestin double-knockout mice and hereditary retinal degeneration caused by the rd6 mutation^19–23^. This effect was also seen even when the main cause of retinal degeneration was not iron dysregulation^22^. Besides, the neuroprotective effects of DFP has been well studied in different neurodegenerative animal models including Alzheimer’s disease, Parkinson’s disease, Huntington’s disease as well as neuronal cultures exposed to different neurotoxic agents^15–18^. Furthermore, a preliminary clinical study reported that the combination of iron depletion and erythropoietin had promising effects in patients with secondary progressive MS^56^.

## Conclusion

The insights gained from this study will advance our understanding of the role of iron deposition and its consequence during the course of demyelination and introduce a new therapeutic approach for treating MS. In summary, iron chelation, using DFP, reduced iron deposition during the course of demyelination, attenuated gliosis and inflammation and preserved myelin, axons and RGCs. The effectiveness of DFP treatment introduce the iron as a novel target for MS treatment, even though further studies are needed to determine the appropriate time window, since iron is required for remyelination and myelin maintenance as well.

## Materials and methods

### Animals

Adult male C57BL/6J mice (8–10-weeks old, body weight 21–25 g) were purchased from Pasteur Institute (Karaj, Iran). Animals were housed under a 12 h light/dark cycle and temperature/humidity-controlled environment with free access to food and water. All experiments were conducted in accordance with the National Institutes of Health (NIH) guidelines for research involving laboratory animals and approved by the Committee of Ethics in Research at Tarbiat Modares University, Tehran, Iran. (Ethical approved ID: IR.MODARES.REC.1398.005). All animal procedures were in accordance with ARRIVE guidelines. Efforts were made to minimize the number of animals used and their suffering.

### Induction of demyelination

Focal demyelination was induced by injection of lysophosphatidylcholine (LPC (lysolecithin), Sigma-Aldrich, L1381), into the optic nerve. Briefly, animals were anesthetized by intraperitoneal (i.p.) injection of ketamine 70 mg/kg (Bremer Pharma GmbH, Germany) and xylazine hydrochloride 10 mg/kg (Hoogstraten, Belgium). Under the guidance of a binocular operating scope (Cambridge Instruments), the conjunctiva was dissected along the glob with Vannas scissors. Fine forceps were used to expose the optic nerve, avoiding damages to retinal blood vessels and excessive stretching to the optic nerve. Demyelination was induced by direct injection of LPC (1 µl, 1%) into the optic nerve 2.5 mm behind the globe, using the Hamilton syringe (Hamilton Company, USA). Tetracycline ointment was applied topically to prevent infections^31,57^.

### Experimental design and treatments

Following deep anesthesia using ketamine and xylazine, animals were scarified at different time points after LPC injection and optic nerve tissues were collected and processed for molecular, biochemical or histological assessments, followed by snap freezing in liquid nitrogen or processing for tissue sectioning. The timelines for histological, biochemical or molecular assessments are shown in Figs. 1A and 2A, respectively. In two additional groups, daily i.p. injections of DFP (Avicenna Laboratories Inc., Saveh, Iran) was started 30 min before LPC injection and continued every 12 h in a 10 mg/kg dose dissolved in 0.9% normal saline, for 3 or 7 continuous days, which are critical time points for investigating inflammation or demyelination, respectively^24^. Vehicle groups received the same volume of saline.

### Tissue processing and sectioning

Following deep anesthesia, animals were sacrificed by transcardial perfusion with phosphate buffer solution (PBS) and 4% paraformaldehyde (PFA). Optic nerve and eyeball were harvested; post-fixed in 4% PFA overnight and protected in 30% sucrose in PBS for 24 h at 4 °C before embedding. The samples were then embedded in optimal cutting medium (OCT; Bio-Optica) followed by freezing at − 20 °C. The blocks were then stored at – 80 °C until sectioning. Using a cryostat (Histo-Line Laboratories, Italy), transverse sections were prepared with 10 μm thickness, starting from the place where the optic nerve merge to the eyeball to the end of optic nerve. Moreover, the posterior eyecup were cut into 10 μm sections. All sections were mounted on superfrost plus slides and stored at − 20 °C for further evaluations.

### DAB enhanced Perl’s staining

DAB enhanced Perl’s staining was used to assess iron deposition in LPC induced demyelination model. The rehydrated cryosections were incubated in 0.3% H2O2 for 10 min in order to quench endogenous peroxidase activity. Then the sections were immersed in fresh-prepared potassium ferrocyanide solution containing equal volume of 5% potassium ferrocyanide and 5% HCl for overnight. HCl releases the ferric iron from the proteins, which allows potassium ferrocyanide to bind to ferric iron. In the presence of H_2_O_2_, this compound catalyzes the oxidative polymerization of 3, 3-diaminobenzidine (DAB), which results in intense dark brown spots^58,59^.

### Hematoxylin and eosin staining

To assess the level of inflammation, Hematoxylin & Eosin (H&E) staining was applied. After rehydration the optic nerve and retina frozen sections were stained with Hematoxylin for 15 min, followed by immersing in Eosin solution. Then, the sections were washed, dehydrated, cleared in xylene and mounted using Entellan (Merck Chemicals, Germany)^24^. A pathologist blinded to the experimental groups evaluated the level of inflammation in different slides (n = 5).

### Myelin staining and immunofluorescence studies

To assess the extent of demyelination, Luxol fast blue (LFB) staining was performed based on previous reports^24^. Briefly, the sections were washed with PBS, incubated in 0.1% LFB for 1 h at 60 °C. To obtained appropriate contrast, the sections were rapidly immersed in 0.05% lithium carbonate, followed by washing in distilled water. Then Cresyl Fast Violet counterstaining was used to visualize cell nuclei. After washing in distilled water, the sections were dehydrated in a graded series of alcohols, cleared in xylene and mounted using entellan (Merck Chemicals, Germany). For quantification, the demyelinated area was calculated as the percentage of total area using Image J software (NIH, USA).

Evaluation of demyelination was done either by FluoroMyelin staining of cryostat sections. After washing with PBS, the sections were incubated in FluoroMyelin (1:300, f34652, ThermoFisher, Oregon, USA) for 30 min at room temperature^60^. The fluorescent intensity was measured by Image J software.

For immunostaining, in brief, frozen sections were incubated in triton-X100, blocking solution, containing NGS 10% (normal goat serum, Sigma) in PBS for 1 h and then incubated with primary antibody at 4 °C overnight. This step was followed by incubation with a corresponding secondary antibody coupled to a fluorescent dye for 2 h at room temperature. After three times of washing with PBS, the sections were counterstained with DAPI (Sigma-Aldrich; D-9542) to visualize cell nuclei^60^. The list of antibodies is provided in Table 1. Myelin basic protein (MBP), Glial fibrillary acidic protein (GFAP), transferrin receptor 1 (Tfr1) and neurofilament 200 (NF-200) intensity was quantified by Image J software. The number of total ionized calcium binding adaptor molecule 1 positive cells (ba1^+^) were presented as the number of cells per section.

**Table 1:** List of primary and secondary antibodies used in this study.

Primary antibody	Label	Species isotype	Supplier	Dilution
MBP	–	Chicken polyclonal	Aves	1:1000
Iba1	–	Rabbit polyclonal	FUJIFILM Wako Pure Chemical	1:1000
GFAP	–	Rabbit polyclonal	Dako	1:500
NF200	–	Chicken polyclonal	Aves	1:100
Tfr1	–	Rabbit polyclonal	Abcam, ab82411	1:500
Anti-chicken IgY	Texas Red	Rabbit	Abcam, ab6751	1:500
Anti-rabbit IgG	Alexa Fluor® 488	Goat	ThermoFisher, A11008	1:1000
Anti-rabbit IgG	Alexa Fluor® 568	Goat	ThermoFisher, A11036	1:1000

All photos were captured under BX-51 fluorescent microscope (Olympus Optical Co, Ltd., Tokyo, Japan). Three to five sections were studied and averaged for each animal group (n = 5 mice per group).

### Malondialdehyde assay

The malondialdehyde (MDA) assay was performed based on a previous reported method with some modifications. In brief, the homogenate samples were mixed with BHT (0.5 mg/ml ethanol) and tricholoroacetic acid (TCA, 25% w/v) solution. The mixture was incubated at 100 °C for 10 min, centrifuged at 2000 rmp for 10 min. This step was followed by mixing the supernatant of each sample with TBA (0.33% w/v) at 95 °C for 1 h. Then the absorbance was measured at 532 nm. Each sample was prepared triplicated for measurements, averaged for each sample then entered into group averaging (n = 4–5 in each time point). Protein concentration of tissue lysate was considered for data normalization^61,62^.

### Glutathione assay

The glutathione (GSH) concentration was measured based on a previous reported method with some modifications^63^. Briefly, the optic nerve samples were homogenized in PBS. Afterward, prepared samples were mixed with the equal volume of 2 mg/ml DTNB (5,5'-dithiobis nitro benzoic acid). The reaction between reduced glutathione and DTNB yield yellow colored product with max absorbance at 412 nm. Each sample was prepared triplicated for measurements and averaged for each sample (n = 4–5 for each time point). Protein concentration of tissue lysate was considered to normalize the readouts.

### Total RNA extraction and real-time polymerase chain reaction

Animals were scarified under deep anesthesia with ketamine and xylazine 1, 2 and 3 days after LPC injection and optic nerve tissues were harvested and snap freezed in liquid nitrogen and stored at − 80 °C until processed. Sample of three animals were pooled for extraction and consequent analysis (totally, 12–15 animals were used for each time point). Total RNA was extracted based on the RNA isolation kit protocol (RiboEx solution, Gene All, Korea). cDNA was reverse-transcribed using cDNA reverse transcription Kit (Parstous Biotechnology, Iran). Afterward, RT-qPCR was performed using SYBR Green PCR Master Mix (Ampliqon, Denmark) on a Rotor-Gene device (Qiagen, Germany). All reactions were performed in duplicate. The relative amount of mRNA was calculated using the 2^−∆∆CT^ method, following standardization relative to GAPDH as a housekeeping gene. The list of the genes and sequences of primers used in this experiment is provided in Table 2.

**Table 2:** Primer sequences used for qPCR.

Gene	Forward sequence	Reverse sequence
*GAPDH*	CATCACTGCCACCCAGAAGACTG	ATGCCAGTGAGCTTCCCGTTCAG
*Fth*	TAAAGAACTGGGTGACCACGTGAC	AAGTCAGCTTAGCTCTCATCACCG
*tfr1*	AAACTGGCTGAAACGGAGGAGACA	GCTGCTTGATGGTGTCAGCAAACT
*Ncoa4*	TTAACACTGCCGACTGGGTT	AGCTGCATACAGGCAAAGAGA
*hspb1*	GATCACTGGCAAGCACGAAGA	CTCGAAAGTAACCGGAATGGTGA
*IREB2*	AGAAACGGACCTGCTCTTCCCA	CCTCTGTCTCAATGCCACCAAC
*GPX4*	CGCAGCCGTTCTTATCAATG	CACTGTGGAAATGGATGAAAGTC
*Acsf2*	TGGAGCAGAAGGCTGGTAGTGT	CATGACGCAGTACCCTCGGATA
*SLC7A11*	CTTTGTTGCCCTCTCCTGCTTC	CAGAGGAGTGTGCTTGTGGACA
*HO-1*	GGTGATGGCTTCCTTGTACC	AGTGAGGCCCATACCAGAAG
*Nrf2*	TCCTATGCGTGAATCCCAAT	GCGGCTTGAATGTTTGTCTT

### Statistical analysis

Statistical analysis was performed using GraphPad Prism 8 software. Shapiro–Wilk test and the Kolmogorov–Smirnov Test were used to assess the normal distribution of data. Multiple comparisons between groups at different time points was analyzed by one-way ANOVA with Dunnett's or Tukey’s multiple comparisons test. Two-tailed unpaired Student's t-test was used in order to compare DFP-treated animals with the vehicle group. *p* values < 0.05 were considered statistically significant. All data are presented as means ± SEM.

## Data Availability

The datasets generated and analyzed during the current study are available from the corresponding author on reasonable request.
